# Antioxidant activities of aqueous extracts from 12 Chinese edible flowers *in vitro* and *in vivo*

**DOI:** 10.1080/16546628.2017.1265324

**Published:** 2016-12-20

**Authors:** Feng Wang, Miao Miao, Hui Xia, Li-Gang Yang, Shao-Kang Wang, Gui-Ju Sun

**Affiliations:** ^a^ Key Laboratory of Environmental Medicine and Engineering of Ministry of Education, Department of Nutrition and Food Hygiene, School of Public Health, Southeast University, Nanjing, China; ^b^ State Key Laboratory of Reproductive Medicine, Department of Nutrition, Nanjing Maternity and Child Health Care Hospital Affiliated to Nanjing Medical University, Nanjing, China

**Keywords:** Edible flowers, antioxidant activity, hypoxia-re-oxygenation, hyperlipidemia

## Abstract

The antioxidant function of edible flowers have attracted increasing interest. However, information is lacking on the impact of edible flowers on oxidative injury including hypoxia-re-oxygenation and hyperlipidemia. The antioxidant activities of aqueous extracts from 12 Chinese edible flowers were assessed in four different antioxidant models, including total antioxidant capacity (TAC), oxygen radical absorbance capacity (ORAC), scavenging hydroxyl radical capacity (SHRC) and scavenging superoxide anion radical capacity (SSARC). Subsequently, the potential antioxidant effects on rat cardiac microvascular endothelial cells (rCMEC) treated with hypoxia-re-oxygenation and hyperlipidemia rats induced by high-fat diet were also evaluated. The highest TAC, ORAC, SHRC and SSARC were *Lonicera japonica* Thunb., *Rosa rugosa* Thunb., *Chrysanthemum indicum* L. and *Rosa rugosa* Thunb., respectively. Most aqueous extracts of edible flowers exhibited good antioxidant effects on injury of rCMEC induced by hypoxia-re-oxygenation. In addition, the aqueous extracts of *Lonicera japonica* Thunb., *Carthamus tinctorius* L., *Magnolia officinalis* Rehd. et Wils., *Rosmarinus officinalis* L. and *Chrysanthemum morifolium* Ramat. could suppress the build-up of oxidative stress by increasing serum superoxide dismutase, glutathion peroxidase, and reducing malonaldehyde concentration in hyperlipidemia rats. These findings provided scientific support for screening edible flowers as natural antioxidants and preventative treatments for oxidative stress-related diseases.

## Abbreviations

GSH-Px: glutathion peroxidase; HDL-C: high density lipoprotein cholesterol; I/R: ischaemia/reperfusion; LDH: lactate dehydrogenase; LDL-C: low density lipoprotein cholesterol; MDA: malonaldehyde; ORAC: oxygen radical absorbance capacity; rCMEC: rat cardiac microvascular endothelial cells; SHRC: scavenging hydroxyl radical capacity; SOD: superoxide dismutase; SSARC: scavenging superoxide anion radical capacity; TAC: total antioxidant capacity; TC: total cholesterol; TG triacylglycerol

## Introduction

There is ample evidence that free radicals, especially reactive oxygen species (ROS), play an essential role in various aspects of physiological and biochemical processes, such as cellular signal transduction, cell proliferation, differentiation and apoptosis [1,2]. However, excessive amount of ROS can produce a myriad of oxidative damages to biomolecules (e.g. lipids, proteins, DNA), which lead to many chronic diseases, including hyperlipidemia, diabetes mellitus, hypertension, ageing and cancer [3–5]. In view of the potential health risks and toxicity of synthetic antioxidants [6,7], naturally effective antioxidants have been widely concerned as preventive and treatment agents.

Many places, such as medieval France and ancient China, have the traditional habit of eating flowers to improve appearance and nutritive value of meals [8,9]. Most edible flowers contain flavonoids, anthocyanins, carotenoids, alkaloids, and many other phenolic compounds, which are rich in antioxidant activity [10–12]. By 2002, about 10 types of Chinese edible flowers have been allowed to be used as either health-care food or medicine by the Ministry of Health of the People’s Republic of China. Recently, scented tea is becoming enormously popular in modern food regimen. Indeed, drinking scented tea has become an indispensable part of Chinese people’s life. Nevertheless, the health effects of edible flowers remain poorly characterized. There is a paucity of information on the impact of edible flowers on oxidative injury including ischaemia/reperfusion (I/R) and hyperlipidemia.

Therefore, the purpose of the present study is to assess antioxidant activities of aqueous extract from 12 Chinese edible flowers, by measuring total antioxidant capacity (TAC), oxygen radical absorbance capacity (ORAC), scavenging hydroxyl radical capacity (SHRC) and scavenging superoxide anion radical capacity (SSARC). In addition, the potential antioxidant effects on rat cardiac microvascular endothelial cells (rCMEC) treated with hypoxia-re-oxygenation and hyperlipidemia rats induced by high-fat diet were also evaluated.

## Materials and methods

### Antioxidant activities assay of edible flowers extracts

A total of 12 dried edible flowers were obtained from Nanjing City in Jiangsu Province, China. Their colour, edible use and traditional medicinal use are presented in Table 1. According to the Pharmacopoeia of the People’s Republic of China [13] and the Flora of China [14], the source of edible flowers was proved by the College of Food Science and Technology, Nanjing Agricultural University, China. The dried flowers were ground into fine particles with a special grinder for food processing. Each sample (2 g) was weighed and mixed with 10 ml double distilled water, then ultrasonic treatment for 30 min. The liquid supernatant were separated through centrifuging at 5000 rpm for 30 min and adjusted to the final volume of 25 ml with double distilled water for antioxidant activities analysis. The TAC, SHRC and SSARC of edible flowers extracts were assayed using commercial kits (Institute of Biological Engineering of Nanjing Jianchen, Nanjing, China) according to the manufacturer’s protocol. The ORAC was determined according to the method of previous assays [15].

**Table 1.** The colour, edible use and traditional medicinal use of edible flowers.

Edible flowers	Colour	Edible use	Traditional medicinal use
*Lonicera japonica* Thunb.	Yellow-green	Tea, soup	Heat-clearing and detoxifying
*Jasminum sambac* (L.) Aiton	White	Tea, porridge	Relieving cough and reducing sputum
*Carthamus tinctorius* L.	Red	Tea, cake	Promoting blood circulation, restoring menstrual flow
*Gardenia jasminoides* Ellis	Reddish brown	Tea, soup	Clearing heat and promoting diuresis
*Magnolia officinalis* Rehd. et Wils.	Reddish brown	Tea	Resolving dampness with aromatics
*Rosa rugosa* Thunb.	Prunosus	Tea, soup	Promoting qi circulation and relieving depression
*Rosmarinus officinalis* L.	Yellow-green	Tea, natural perfume	Tranquilizing and allaying excitement
*Chrysanthemum indicum* L.	Brown-yellow	Tea, cake	Heat-clearing and detoxifying
*Chrysanthemum morifolium* Ramat.	Yellow-white	Tea, cake	Heat-clearing and detoxifying
*Eugenia caryophyllata* Thunb.	Dark brown	Tea	Warming middle energizer descend adverse-rising
*Sophora japonica* L.	Yellow	Tea, cake	Cooling blood and haemostasis
*Myosotis silvatica* Ehrh. ex Hoffm.	Blue	Tea	Heat-clearing and detoxifying

### Edible flowers extract preparation for cell and animal assay

Each sample (300 g) was weighed and extracted with 2000 ml boiling distilled water for 1 hour. After cooling the extracts were filtered using a filter paper, and then the filtrates were diluted to a solution of 0.3 g/ml and stored at 4°C for further analysis *in vitro* and *in vivo*.

### Isolation of primary rCMEC

The rCMEC were isolated as described in previous report [16]. Briefly, the Wistar rats (7–10 days) hearts were rapidly removed, rinsed, and bathed with 75% alcohol in order to inactivate endothelial cells in epicardium and endocardium. Hearts were minced and re-suspended with 2 ml 0.2% type II collagenase, then incubated for 30 min with 37°C. They were digested for 10 min with 0.02% pancreatic enzyme solution. After digestion, the cells were filtered through sterile nylon mesh, and centrifuged at 1000 rpm for 5 min. The cell pellet was re-suspended in 8 ml Dulbecco’s modified Eagle’s medium (DMEM, Life Technologies/Gibco, Grand Island, NY) containing 10% foetal calf serum (FCS, Life Technologies/Gibco, Grand Island, NY), 100 U/ml penicillin and 100 µg/ml streptomycin (Life Technologies/Gibco, Gaithersburg, MD) and plated into culture flasks treated with 1% gelatin. Non-adherent cells were removed after 37°C for 4 h and discarded. The adherent cells were rCMEC. These protocols were approved by the Institutional Animal Care and Use Committee of Southeast University.

### Hypoxia and re-oxygenation of primary rCMEC

For hypoxia and re-oxygenation treatment, rCMEC were cultured in serum-free DMEM medium supplemented with 0.5 mol/L de-oxidant sodium dithionite in 37°C with 5% v/v CO_2_ incubator for 1 h. Then, the cells were maintained in DMEM medium supplemented with 10% foetal calf serum in 37°C with 5% v/v CO_2_ incubator for another 1 h.

### Cell proliferation and apoptosis assay

The rCMEC (2.5 × 10^3^/well) were seeded in 96-well plates and divided into 14 groups, including control group (normal cells), model group (hypoxia 1 h then re-oxygenation 1 h) and model group plus 12 edible flowers extracts (12 groups, respectively). The cells were treated with 750 μg/L edible flowers extracts in DMEM for 24 h then treated with hypoxia for 1 h then re-oxygenation for 1 h. Cell proliferation was measured using MTT method.

The rCMEC (2 × 10^5^/well) were seeded in 24-well plates and treated in accordance with methods described above. The cell apoptosis was assessed by a flow cytometric analysis using propidium iodide (PI) (Sigma, USA) staining according to the manufacturer’s protocol. Briefly, the cells were collected via trypsinization and washed with phosphate-buffered saline (PBS). The cells were fixed with 95% acetic acid and stored at 4°C overnight. The following day, the cells were washed with PBS, treated with RNase A (50 μg/ml), and stained with propidium iodide (PI) (50 μg/ml) for 30 min in the dark. The cells were subsequently analyzed via flow cytometry (CoulterEPICS-XL System II).

### Cell antioxidant activities assay

The rCMEC were divided into 14 groups, including control group (normal cells), model group (hypoxia 1 h then re-oxygenation 1 h) and model group plus 12 edible flowers extracts (12 groups, respectively). The cell culture supernatant were collected and the levels of lactate dehydrogenase (LDH), malonaldehyde (MDA) and superoxide dismutase (SOD) were assayed using commercial kits (Nanjing Jiancheng Bioengineering Institute, Nanjing, China) according to the manufacturer’s protocol.

### Animal treatment

The animal experiment protocols used in this study were reviewed and approved by the Institutional Animal Care and Use Committee of Southeast University. A total of 140 male Wistar rats weighing 90–110 g were purchased from the Shanghai Experimental Animal Center of Chinese Academy of Sciences (Shanghai, China). All animals were kept under controlled temperature (20–25°C) and on a 12-h light/12-h dark cycle. After an adaptation period of 7 days, the Wistar rats were starved for 12 h and were tested the level of total cholesterol (TC) in the serum. Based on corresponding TC level and body weight, rats were randomly divided into 14 groups (*n *= 10 in each group), including chow diet group, high-fat diet group and high-fat diet plus 12 edible flowers extracts (12 groups, respectively). The food composition of the diets is shown in Table 2. Twelve edible flowers extracts were administered by gavage once a day at a dose of 3 g/kg bw. The chow diet and high-fat diet group were administered by gavage with the equivalent of distilled water. The diet and edible flowers extracts treatment lasted for 6 weeks.

**Table 2.** Diet composition of animal experiment.

Component (weight %)	Chow diet	High-fat diet
Casein	23.0	20.0
Maize starch	32.0	29.0
Sucrose	31.0	25.3
Cellulose	4.0	4.0
Colza oil	5.0	0.0
Lard	0.0	10.0
Egg yolk powder	0.0	5.0
Cholesterol	0.0	1.5
Bile salts	0.0	0.2
Mineral mix (AIN-76A)	3.5	3.5
Vitamin mix (AIN-76A)	1.0	1.0
DL-Methionine	0.3	0.3
Choline chloride	0.2	0.2
Total	100.0	100.0

### Serum antioxidant capacities assay and lipid profiles

At the end of 6 weeks, the rats were fasted overnight and were anaesthetized with sodium pentothal. The blood samples were collected from the abdominal aorta. Then the serum samples were separated through centrifuging at 3000 rpm for 10 min. The serum MDA, SOD and glutathion peroxidase (GSH-Px) were measured using commercial kits (Nanjing Jiancheng Bioengineering Institute, Nanjing, China) according to the manufacturer’s protocol. Additionally, the serum TC, triacylglycerol (TG), high density lipoprotein cholesterol (HDL-C) and low density lipoprotein cholesterol (LDL-C) were also assayed using commercial kits (Nanjing Jiancheng Bioengineering Institute, Nanjing, China) according to the manufacturer’s protocol.

### Statistical analysis

Each value was expressed as means ± SD. Data were analysed using One-way Analysis of Variance (ANOVA). All the analyses were performed in PASW statistics 18.0 (SPSS Inc, Chicago, IL, USA). p value less than 0.05 has been considered significant.

## Results

### Antioxidant activities of edible flowers extracts

As observed in Table 3, four different antioxidant models, including TAC, ORAC, SHRC and SSARC were used to evaluate the antioxidant activities of edible flowers extracts. *Lonicera japonica* Thunb. possessed the highest TAC, followed by *Rosmarinus officinalis* L., *Chrysanthemum indicum* L. and *Myosotis silvatica* Ehrh. ex Hoffm. *Jasminum sambac* (L.) Aiton is characterized by the lowest TAC, which is 4.3-fold lower than *Lonicera japonica* Thunb. The ORAC ranged from 46.11 ± 0.10 μmol TE/g (*Jasminum sambac* (L.) Aiton) to 861.87 ± 0.25 μmol TE/g (*Rosa rugosa* Thunb.). *Chrysanthemum indicum* L., *Myosotis silvatica* Ehrh. ex Hoffm. and *Chrysanthemum morifolium* Ramat. exhibited the higher SHRC with more than 1100.00 U/g. The lowest SHRC was found in *Rosa rugosa* Thunb., *Rosa rugosa* Thunb., *Rosmarinus officinalis* L. and *Chrysanthemum morifolium* Ramat. showed the higher SSARC when compared with other extracts. The lowest SSARC was revealed only in *Gardenia jasminoides* Ellis with value of 103.02 U/g.

**Table 3.** Antioxidant activities of edible flowers extracts *in vitro*.

Edible flowers	TAC(U/g)	ORAC(μmol TE/g)	SHRC(U/g)	SSARC(U/g)
*Lonicera japonica* Thunb.	2205.2 ± 4.6	138.84 ± 0.26	969.1 ± 6.3	625.0 ± 0.8
*Jasminum sambac* (L.) Aiton	517.1 ± 3.1	46.11 ± 0.10	1083.3 ± 0.7	723.4 ± 4.1
*Carthamus tinctorius* L.	885.1 ± 5.5	67.38 ± 0.12	1020.3 ± 3.8	636.5 ± 5.3
*Gardenia jasminoides* Ellis	1085.9 ± 5.6	60.72 ± 0.22	965.1 ± 3.0	103.0 ± 2.0
*Magnolia officinalis* Rehd. et Wils.	540.6 ± 2.0	95.85 ± 0.40	1028.2 ± 5.2	760.1 ± 3.7
*Rosa rugosa* Thunb.	720.9 ± 3.7	861.87 ± 0.25	724.9 ± 1.3	986.7 ± 2.7
*Rosmarinus officinalis* L.	2077.5 ± 7.9	296.91 ± 0.20	984.8 ± 2.2	973.0 ± 6.8
*Chrysanthemum indicum* L.	1499.5 ± 2.9	57.42 ± 0.14	1110.9 ± 2.8	666.2 ± 3.1
*Chrysanthemum morifolium* Ramat.	1174.1 ± 6.1	76.32 ± 0.30	1103.0 ± 3.0	924.9 ± 3.5
*Eugenia caryophyllata* Thunb.	1940.6 ± 1.4	310.23 ± 0.19	819.4 ± 4.7	615.8 ± 1.2
*Sophora japonica* L.	763.5 ± 1.8	174.36 ± 0.21	1051.8 ± 9.8	709.7 ± 1.1
*Myosotis silvatica* Ehrh. ex Hoffm.	1752.0 ± 2.7	98.94 ± 0.30	1107.0 ± 4.9	783.0 ± 1.7

Values represent the means ± SD of 3 for each group.

TAC, total antioxidant capacity; ORAC, oxygen radical absorbance capacity; SHRC, scavenging hydroxyl radical capacity; SSARC, scavenging superoxide anion radical capacity.

### Effects of edible flowers extracts on rCMEC proliferation and apoptosis

Upon the hypoxia 1 h then re-oxygenation 1 h treatment, there was a large decrease in the proliferation of rCMEC. The cells pre-treated with edible flowers extracts could attenuate this damage, especially in groups of *Carthamus tinctorius* L., *Chrysanthemum indicum* L. and *Chrysanthemum morifolium* Ramat (Table 4). We then selected the three above edible flowers extracts to measure the cell apoptosis by a flow cytometric analysis. The results showed that the cells apoptosis increased significantly in the hypoxia-re-oxygenation treated group, and a certain degree of decreased cell apoptosis were found in edible flowers extracts group, especially in *Chrysanthemum morifolium* Ramat (Table 4).

**Table 4.** Effects of edible flowers extracts on rCMEC proliferation and apoptosis.

Group	Proliferation	Apoptosis (%)
Normal cell	63.57 ± 0.83	4.58 ± 0.41
Hypoxia-re-oxygenation cell	24.89 ± 1.21^a^	54.04 ± 1.91^a^
*Lonicera japonica* Thunb.	22.74 ± 0.67	Nd
*Jasminum sambac* (L.) Aiton	26.60 ± 1.14	Nd
*Carthamus tinctorius* L.	45.92 ± 0.72^b^	29.06 ± 1.18^b^
*Gardenia jasminoides* Ellis	34.76 ± 1.33^b^	Nd
*Magnolia officinalis* Rehd. et Wils.	28.75 ± 0.96^b^	Nd
*Rosa rugosa* Thunb.	15.02 ± 1.31	Nd
*Rosmarinus officinalis* L.	28.32 ± 0.89^b^	Nd
*Chrysanthemum indicum* L.	52.78 ± 1.02^b^	29.88 ± 1.27^b^
*Chrysanthemum morifolium* Ramat.	51.93 ± 0.93^b^	5.04 ± 0.27^b^
*Eugenia caryophyllata* Thunb.	14.16 ± 0.76	Nd
*Sophora japonica* L.	39.05 ± 1.34^b^	Nd
*Myosotis silvatica* Ehrh. ex Hoffm.	32.61 ± 1.32^b^	Nd

Values represent the means ± SD of 5 for each group, ^a^ *P *< 0.05 vs. Normal cell group, ^b^ *P *< 0.05 vs. Hypoxia-re-oxygenation cell group.

Nd, not detect.

### Antioxidant effects of edible flowers extracts on rCMEC induced by hypoxia-re-oxygenation

In response to hypoxia-re-oxygenation, concentrations of circulating enzyme antioxidants such as SOD decreased, whereas LDH and MDA increased in the hypoxia-re-oxygenation cells group compared with the normal cell group (Table 5). Generally, most edible flowers extracts, with a few exceptions (like *Myosotis silvatica* Ehrh. ex Hoffm.), exhibited good antioxidant activities.

**Table 5.** Antioxidant effects of edible flowers extracts on rCMEC induced by hypoxia-re-oxygenation.

Group	LDH (U/L)	MDA (nmol/ml)	SOD (U/ml)
Normal cell	138.80 ± 11.25	1.02 ± 0.08	51.61 ± 3.43
Hypoxia-re-oxygenation cell	191.37 ± 15.37^a^	2.89 ± 0.22^a^	17.95 ± 1.23^a^
*Lonicera japonica* Thunb.	172.59 ± 7.61^b^	2.47 ± 0.20	25.57 ± 3.60^b^
*Jasminum sambac* (L.) Aiton	176.59 ± 7.61^b^	2.58 ± 0.37	26.39 ± 5.41^b^
*Carthamus tinctorius* L.	158.52 ± 9.71^b^	2.04 ± 0.22^b^	30.85 ± 5.35^b^
*Gardenia jasminoides* Ellis	168.37 ± 10.03^b^	2.37 ± 0.31^b^	23.83 ± 5.49^b^
*Magnolia officinalis* Rehd. et Wils.	169.19 ± 9.73^b^	2.11 ± 0.18^b^	28.29 ± 3.22^b^
*Rosa rugosa* Thunb.	158.52 ± 8.76^b^	2.55 ± 0.24^b^	19.66 ± 1.19
*Rosmarinus officinalis* L.	166.73 ± 9.91^b^	2.35 ± 0.18^b^	27.14 ± 7.08^b^
*Chrysanthemum indicum* L.	155.23 ± 13.14^b^	1.97 ± 0.32^b^	32.30 ± 4.87^b^
*Chrysanthemum morifolium* Ramat.	151.12 ± 8.46^b^	1.93 ± 0.21^b^	34.32 ± 5.58^b^
*Eugenia caryophyllata* Thunb.	167.55 ± 12.23^b^	2.88 ± 0.29	22.64 ± 4.36^b^
*Sophora japonica* L.	160.98 ± 9.14^b^	2.47 ± 0.19^b^	23.32 ± 3.32^b^
*Myosotis silvatica* Ehrh. ex Hoffm.	188.09 ± 10.27	2.62 ± 0.11	21.53 ± 3.97^b^

Values represent the means ± SD of 5 for each group, ^a^ *P *< 0.05 vs. Normal cell group, ^b^ *P *< 0.05 vs. Hypoxia-re-oxygenation cell group.

LDH, lactate dehydrogenase; MDA, malonaldehyde; SOD, superoxide dismutase.

### Antioxidation and lipid regulation of edible flowers extracts in hyperlipidemia rats

In the present study, the serum MDA, TC, TG and LDL-C were increased, whereas SOD, GSH-Px and HDL-C were decreased in the experimental rats fed with high-fat diets for 6 weeks compared with those in the chow diet group (Table 6). Nevertheless, the serum MDA level was significantly declined in the groups of *Lonicera japonica* Thunb., *Carthamus tinctorius* L., *Gardenia jasminoides* Ellis, *Magnolia officinalis* Rehd. et Wils., *Rosmarinus officinalis* L., *Chrysanthemum indicum* L., *Chrysanthemum morifolium* Ramat. and *Sophora japonica* L.; the serum SOD and GSH-Px were significantly increased in the groups of *Lonicera japonica* Thunb., *Carthamus tinctorius* L., *Magnolia officinalis* Rehd. et Wils., *Rosmarinus officinalis* L., *Chrysanthemum morifolium* Ramat. and *Eugenia caryophyllata* Thunb. The aqueous extracts of *Gardenia jasminoides* Ellis and *Magnolia officinalis* Rehd. et Wils. could suppress the build-up of hyperlipidemia by reducing serum TC, TG and LDL-C, and increasing serum HDL-C.

**Table 6.** Antioxidation and lipid regulation of edible flowers extracts in hyperlipidemia rats.

Group	MDA (nmol/ml)	SOD (U/ml)	GSH-Px (U/ml)	TC (mmol/L)	TG (mmol/L)	LDL-C (mmol/L)	HDL-C (mmol/L)
Chow diet	3.74 ± 0.20	175.48 ± 7.72	675.48 ± 235.38	2.82 ± 0.50	1.84 ± 0.71	2.73 ± 0.49	1.89 ± 0.49
High-fat diet	5.58 ± 1.24^a^	154.46 ± 8.17^a^	288.38 ± 241.10^a^	5.20 ± 1.39^a^	4.02 ± 1.03^a^	5.04 ± 1.38^a^	0.87 ± 0.25^a^
*Lonicera japonica* Thunb.	4.78 ± 0.64^b^	164.45 ± 5.56^b^	504.68 ± 248.49^b^	4.74 ± 0.81	2.99 ± 1.14^b^	4.62 ± 0.80	0.95 ± 0.21
*Jasminum sambac* (L.) Aiton	5.04 ± 1.12	157.13 ± 7.91	349.83 ± 82.56	5.03 ± 0.63	2.74 ± 0.92^b^	4.93 ± 0.63	1.16 ± 0.37^b^
*Carthamus tinctorius* L.	4.11 ± 0.77^b^	160.89 ± 5.92^b^	630.48 ± 291.26^b^	4.72 ± 0.76	2.97 ± 1.52^b^	4.62 ± 0.77	1.16 ± 0.20^b^
*Gardenia jasminoides* Ellis	4.80 ± 1.36^b^	161.48 ± 7.35^b^	453.39 ± 240.53	4.31 ± 1.08^b^	2.70 ± 0.91^b^	4.22 ± 1.09^b^	1.40 ± 0.17^b^
*Magnolia officinalis* Rehd. et Wils.	4.37 ± 0.89^b^	160.72 ± 8.82^b^	566.61 ± 96.92^b^	4.33 ± 0.83^b^	2.67 ± 0.71^b^	4.23 ± 0.83^b^	1.48 ± 0.38^b^
*Rosa rugosa* Thunb.	4.92 ± 0.83	159.85 ± 6.02	339.19 ± 174.24	4.89 ± 1.17	3.74 ± 0.99	4.78 ± 1.18	0.91 ± 0.13
*Rosmarinus officinalis* L.	4.71 ± 0.78^b^	161.68 ± 6.10^b^	668.23 ± 210.24^b^	4.30 ± 0.52^b^	3.25 ± 0.51	4.23 ± 0.52^b^	1.49 ± 0.35^b^
*Chrysanthemum indicum* L.	3.75 ± 0.67^b^	159.99 ± 7.29	590.32 ± 147.35^b^	4.25 ± 0.70^b^	2.93 ± 1.44^b^	4.16 ± 0.71^b^	1.05 ± 0.22
*Chrysanthemum morifolium* Ramat.	3.95 ± 0.32^b^	163.37 ± 2.51^b^	665.81 ± 149.37^b^	4.43 ± 0.84	4.28 ± 1.55	4.34 ± 0.83	0.94 ± 0.12
*Eugenia caryophyllata* Thunb.	4.93 ± 0.82	159.85 ± 6.02^b^	493.55 ± 289.48^b^	5.45 ± 1.21	3.73 ± 1.39	5.33 ± 1.22	1.01 ± 0.24
*Sophora japonica* L.	4.20 ± 0.29^b^	158.20 ± 5.83	456.29 ± 222.67	5.18 ± 0.92	3.83 ± 1.00	5.07 ± 0.93	1.18 ± 0.24^b^
*Myosotis silvatica* Ehrh. ex Hoffm.	5.25 ± 0.79	156.20 ± 6.76	329.52 ± 219.96	5.30 ± 0.94	4.14 ± 1.38	5.17 ± 0.93	1.15 ± 0.40^b^

Values represent the means ± SD of 10 for each group, ^a^ *P *< 0.05 vs. Chow diet group, ^b^ *P *< 0.05 vs. High-fat diet group.

MDA, malonaldehyde; SOD, superoxide dismutase; GSH-Px, glutathion peroxidase; TC, total cholesterol, TG, triacylglycerol;

HDL-C, high density lipoprotein cholesterol; LDL-C, low density lipoprotein cholesterol.

## Discussion

There are so many edible flowers all over the world that only a small part of them have been studied for their antioxidant capacities. Furthermore, little is known about the impact of edible flowers on oxidative injury including hypoxia-re-oxygenation and hyperlipidemia. In the present study, the antioxidant activities of aqueous extracts from 12 Chinese edible flowers were assessed in four different methods. Subsequently, the potential antioxidant effects on rCMEC treated with hypoxia-re-oxygenation and hyperlipidemia rats induced by high-fat diet were also evaluated.

We observed that *Rosa rugosa* Thunb. had the highest antioxidant capacity in both ORAC and SSARC, which was in agreement with the published studies [17–19]. Ng et al. reported the major antioxidant principles of the aqueous extract of *Rosa rugosa* Thunb. were associated with the presence of both gallic acid derivatives and polysaccharides [20]. In addition, the significance of antioxidant activity in *Lonicera japonica* Thunb. and *Chrysanthemum indicum* L. was also mentioned, for instance by Zeng et al. [21]. More recently, ethanol extracts of eight edible flowers collected in Italy were analysed for their antioxidant activities. The results show that edible flowers can serve as natural antioxidants [22]. An important thing to note is that the antioxidant effects are different based on the various extracts from edible flowers. Liu et al. found that the antioxidant capacities of the *Litchi chinenesis* Sonn. extracts were in a descending order as: acetone extract, methanol extract and water extract [23]. Rather, the methanol extract of *Rosmarinus officinalis* L. had higher antioxidant activity than its ethanol and water extract [24]. In our investigation, we only determined antioxidant activities of aqueous extract from edible flowers. This extraction is consistent with the way to have scented tea, which can be guidance for development and application of beverages made from edible flowers. However, it should be mentioned that the antioxidant activity *in vitro* might be regulated by food digestion and metabolism in the diet.

I/R is a process that restores the blood flow after temporary hypoxia, and its main underlying mechanisms involve oxidative injury [25]. During the reperfusion stage, large amounts of oxygen inflowing with nutritive blood activates the hypoxanthine-xanthine oxidase system, causing overproduction of superoxide anion free radicals [26]. Although the hypoxia-re-oxygenation injury in rCMEC is one of the most important models in explaining the mechanisms involved in I/R [27], their nutraceutical application is little-studied, particularly research in edible flowers. In this study, we isolated the primary rCMEC and found that edible flowers extracts can protect rCMEC against apoptosis and alleviate impairment induced by hypoxia-re-oxygenation. Supporting our results, a previous study reported that *Hibiscus sabdariffa* L. extract reduce intracellular reactive oxygen species formation of primary vascular endothelial cells and improve cell viability, following oxidative stress in dose-dependent manner [28]. At present, it is clear that these health benefits are attributed to the antioxidant activity derived from the high level of phenolic compounds present in edible flowers [15]. Besides, quercetin-3-rhamnoglucoside (rutin) [29], chlorogenic acid and caffeic acid [30] play a major role in the protective effect on I/R injury. Identification and quantification of phenolic compounds, however, are not addressed in our study. These questions are worthy of in-depth study.

High-fat diet is the direct source of oxidative stress and hyperlipidemia [31], which play critical roles in the pathogenesis of cardiovascular disease [32,33]. To counteract the oxidants, the antioxidant system attempts to boost endogenous antioxidants to protect cells from oxidative damage, which includes antioxidant enzymes such as SOD and GSH-Px [34,35]. As expected, the present study has shown that the aqueous extracts of *Lonicera japonica* Thunb., *Carthamus tinctorius* L., *Magnolia officinalis* Rehd. et Wils., *Rosmarinus officinalis* L. and *Chrysanthemum morifolium* Ramat. inhibit the increase in oxidative stress in high-fat diet group, characterized by the decrease of serum MDA content and the elevation of SOD and GSH-Px levels. The amount of total phenolic content and total flavonoid content seems to be responsible for much of this edible flower’s efficacy as natural antioxidants [36,37]. As far as *Chrysanthemum morifolium* Ramat. are concerned, Luteolin 7-O-(6″O-malonyl)-glucoside is a predominant flavonoid with the strongest radical scavenging activity [38]. Furthermore, it was suggested that compounds with chemical structures such as Luteolin 7-O-(6″O-malonyl)-glucoside have preventive effects against liver injury in mice induced by injection of carbon tetrachloride [39]. These results warrant further research, particularly studies dedicated to the elucidation of unidentified compounds which likely contribute to the favourable effects on antioxidant status.

Recent advances in edible flowers research have expanded the evidence for the role in modulating cardiometabolic risk factors [40,41]. These studies suggest that edible flowers extracts have a beneficial effect on hyperlipidemia risk factors by enhancing favorable lipid profiles. Furthermore, the flower of *P. notoginseng and P. pashia* have been used as traditional herbal medicine for regulating the lipid of blood in China [42,43]. In the present study, as far as *Gardenia jasminoides* Ellis and *Magnolia officinalis* Rehd. et Wils. are concerned, they show not only an antioxidant but also a marked inhibiting effects on hyperlipidemia. Unfortunately, the precise mechanisms for how edible flowers extract improved serum lipid profiles in hyperlipidemia rats were not explored. The improvement of lipid-increasing action might be accounted for by the unique nutrient composition of the edible flowers extracts.

Despite growing interest in the protective role that edible flowers may have in the development of oxidative injury and hyperlipidemia, little evidence is available in the toxicology of edible flowers. The most recent review reported that edible flowers were non-toxic at an appropriate dosage [44]. For example, an acute toxicity study in a rats model revealed that a single oral dose of 15 g/kg bw ethanolic extract of *Chrysanthemum morifolium* Ramat. from china exhibited no observed adverse effect, and similar results were also found in the long-term toxicity study [45]. Yoo et al. found that 0.1, 0.5, 1.0 mg/ml ethanol extract of *Lonicera japonica* Thunb. showed no cytotoxicity in RAW264.7 macrophage cells [46]. Due to the limitations of the available evidence, however, further studies are warranted.

Our study had several limitations. First, different extraction methods make it difficult to investigate the relationship between antioxidant activities of edible flowers extracts and their antioxidant effects on rCMEC treated with hypoxia-re-oxygenation and hyperlipidemia rats induced by high-fat diet. Second, we did not further define the details of bioactivity responsible for the observed effects. Finally, only a single dose group is offered, so the dose–effect relationship has yet to be described. In the next study, in order to better understand how antioxidant effects of edible flowers work, it is necessary to identify and isolate the active compounds of edible flowers. The underlying antioxidant mechanisms and potential side effects of edible flowers also warrant further exploration and experimentation.

## Conclusions

In this study, the antioxidant activities of aqueous extracts from 12 Chinese edible flowers were systematically evaluated by various methods *in vitro* and *in vivo*. These results indicated that the aqueous extracts of *Rosmarinus officinalis* L. and *Chrysanthemum morifolium* Ramat. possess good antioxidant effects both *in vitro* and *in vivo*. Furthermore, *Gardenia jasminoides* Ellis and *Magnolia officinalis* Rehd. et Wils. extracts markedly improved lipid metabolism in hyperlipidemia rats. Our findings highlight experimental evidence from cell models and animal-based studies that have elucidated the effects of edible flowers on the antioxidant effects and its potential application in strategies to improve oxidative stress-related diseases.
